# Recurrent Necrotizing Fasciitis: A Case Report of Fulminant and Sub-Acute Necrotizing Fasciitis in a Diabetic Patient

**DOI:** 10.7759/cureus.12153

**Published:** 2020-12-18

**Authors:** Johannes Peters, Jean Iacobelli, Emily Ryan

**Affiliations:** 1 General Surgery, Royal Perth Hospital, Perth, AUS; 2 Pathology, Fiona Stanley Hospital, Perth, AUS; 3 Plastic and Reconstructive Surgery, Royal Perth Hospital, Perth, AUS

**Keywords:** necrotizing infection, necrotizing fasciitis

## Abstract

Necrotizing fasciitis is an uncommon and deadly disease entity characterized by rapidly progressing skin and soft tissue destruction. It presents on a spectrum from an initially indolent appearing sub-acute form to a hyperacute fulminant course. It may often be misdiagnosed due to the paucity of signs early in the disease course and as it can initially mimic other less serious soft tissue infections. Necrotizing soft tissue infections have both high morbidity and mortality.

We present a case of a 72-year-old male patient with two anatomically and temporally separate necrotizing infections. The first necrotizing infection was diagnosed after an extended time, due to the subacute disease course in the setting of an abdominal wall infection. The second presentation was a hyperacute fulminant course in the setting of a necrotizing infection of the scrotum. In both instances, once identified, appropriate management was followed: resuscitation, broad-spectrum antibiotics, and most importantly radical surgical debridement. Extensive multidisciplinary inpatient and outpatient input was required to aid the patient’s recovery.

The presented case demonstrates the necrotizing soft tissue infection’s spectrum of disease and the diagnostic dilemma it presents to family physicians and emergency departments alike. The only definitive management step is immediate and radical resection of the affected tissue. Extensive debridement and the resultant tissue defect require comprehensive multidisciplinary care during the extended rehabilitation and wound care treatment plan. Rapid recognition, urgent surgical debridement, and specialist care are required to reduce the mortality and morbidity associated with necrotizing soft tissue infections.

## Introduction

Necrotizing soft tissue infection (NSTI), also known as necrotizing fasciitis, is an uncommon disease entity that is characterised by rapidly progressing skin and soft tissue destruction [1,2] with a high mortality rate. Early radical surgical resection of infected tissue coupled with broad-spectrum antibiotic coverage and systemic support for sepsis are imperative to improve survival [1-3]. It is proposed that NSTIs [4-7] exist on a continuum and range from subacute to hyperacute fulminant infections. Sub-acute NSTIs are indolent and of a protracted time course eventually resulting in an acute deterioration. This case report details a patient with two anatomically and temporally separate presentations of a necrotizing infection, exhibiting different manifestations of the disease.

## Case presentation

The patient is a 72-year-old male with a history of poorly controlled type 2 diabetes mellitus, chronic renal disease secondary to diabetic nephropathy, extensive lifetime smoking history, and morbid obesity.

His initial presentation (day 1) occurred after a two-week history of lower abdominal pain, associated skin breakdown and erythema of his abdominal pannus which was initially treated by the family physician as an intertrigo/uncomplicated cellulitis. Over a two-week period the patient began to report systemic symptoms including nausea, vomiting and chills. On presentation to the emergency department in a tertiary referral hospital, the skin under his abdominal pannus was indurated and erythematous. Additionally, a fluctuant mass with purple discolouration approximately 10cm in diameter, discharging offensive purulent fluid was noted. No abnormal systemic signs were present on physical examination. Inflammatory markers were elevated, in addition to severe hyperglycaemia, an acute on chronic kidney injury was noted and a wound swab grew penicillin-sensitive *Streptococcus anginosus* (formerly *S. milleri*) (Table 1).

**Table 1:** Patient investigation results and LRINEC scores throughout hospital admission eGFR: Estimated glomerular filtration rate LRINEC: Laboratory risk indicator for necrotizing soft tissue infections scoring criteria

Investigation	Day 1	Day 12	Day 114	Day 142	Day 169
Hemoglobin (norm: 135-180g/L)	127	104	119	104	94
White cell count (norm: 4-11x10^9/L )	14.4	<11	11.1	14.9	9.4
Serum C reactive protein (norm: <5mg/L)	310	22	55	350	16
Serum sodium (norm: 135-145 mmol/L)	125	139	133	130	138
Serum creatinine (norm 60-100µmol/L)	306	143	167	287	170
eGFR (norm: >60mL/min/1.73m^2 )	17	43	35	18	34
Serum glucose (norm: 3.0-5.4 mmol/L)	21.8	normal range	9.9	18.5	normal range
LRINEC score	10	<6	<6	12	<6

The initial diagnosis of abdominal wall abscess and cellulitis was made but a high suspicion for NSTI persisted due to his aforementioned risk factors. Over the subsequent hours he deteriorated and became critically unwell. Fluid resuscitation was started and he was commenced on an insulin dextrose infusion and broad-spectrum antibiotics according to local antimicrobial guidelines in addition to emergent operating theatre access.

**Figure 1:**
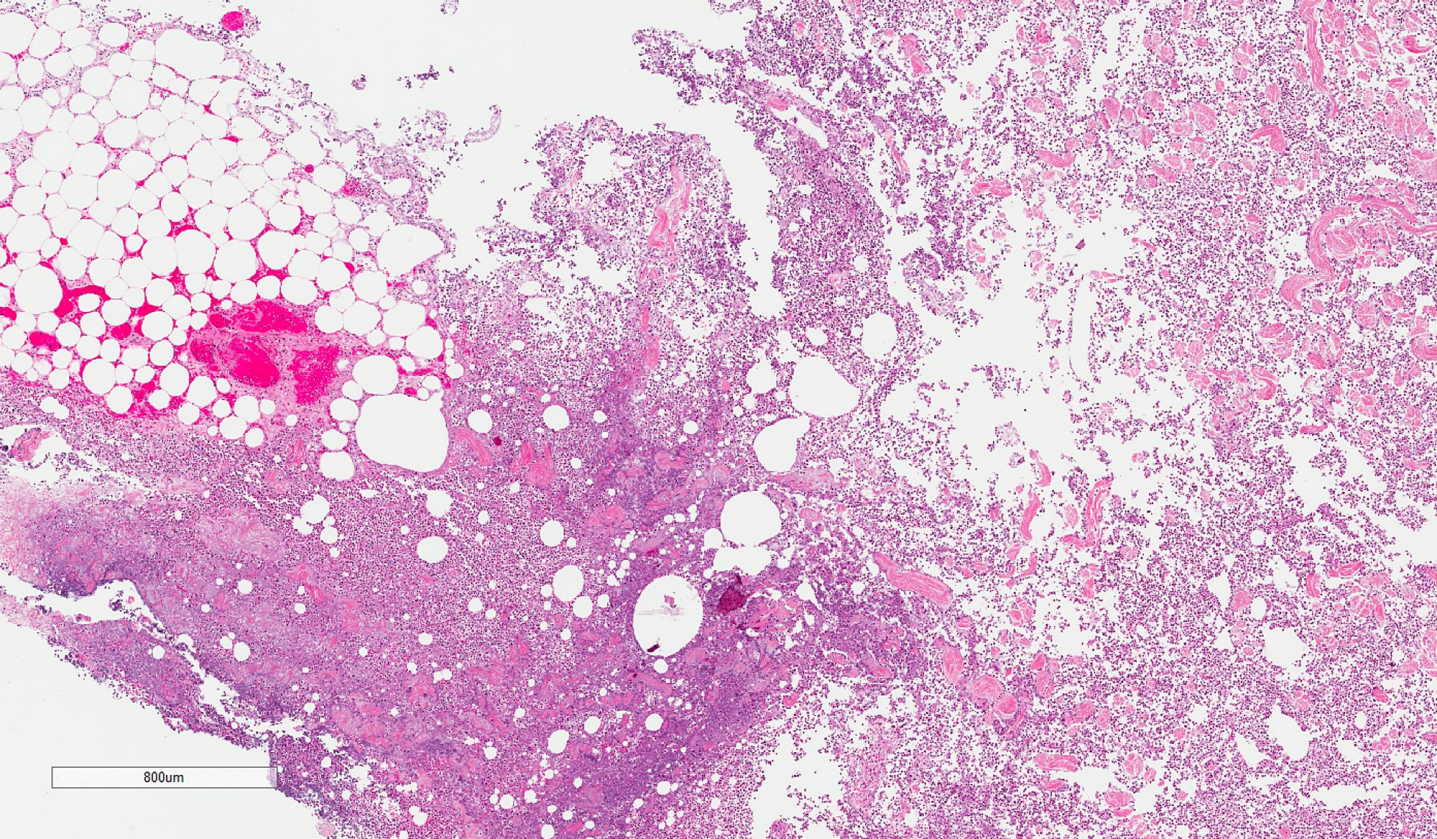
Abdominal wall- Deep subcutaneous tissue showing diffuse neutrophil rich inflammation with suppuration and associated necrosis extending through the full thickness of the biopsy (H&E 20x)

An aggressive excision of necrotic skin and fat occurred with debridement to healthy tissue. No peritoneal involvement was observed. Histopathology demonstrated findings consistent with necrotizing fasciitis (Figure 1). A second debridement occurred on day 3 and the patient was discharged on day 12 with a vacuum-assisted closure (VAC) device, normalised inflammatory markers and return to baseline renal function (Table 1). Wounds healed by secondary intention over the course of four months.

Subsequently the patient represented on day 24 for minor debridement and reapplication of VAC device dressing. Day 63 for conservative management of wound dehiscence after coughing fit, with no signs of infection and normal blood work. Around day 80 the patient’s family physician noted completed healing of the abdominal wound. Day 114 another debridement occurred due to abscess formation, but no tissue necrosis was noted.

**Figure 2:**
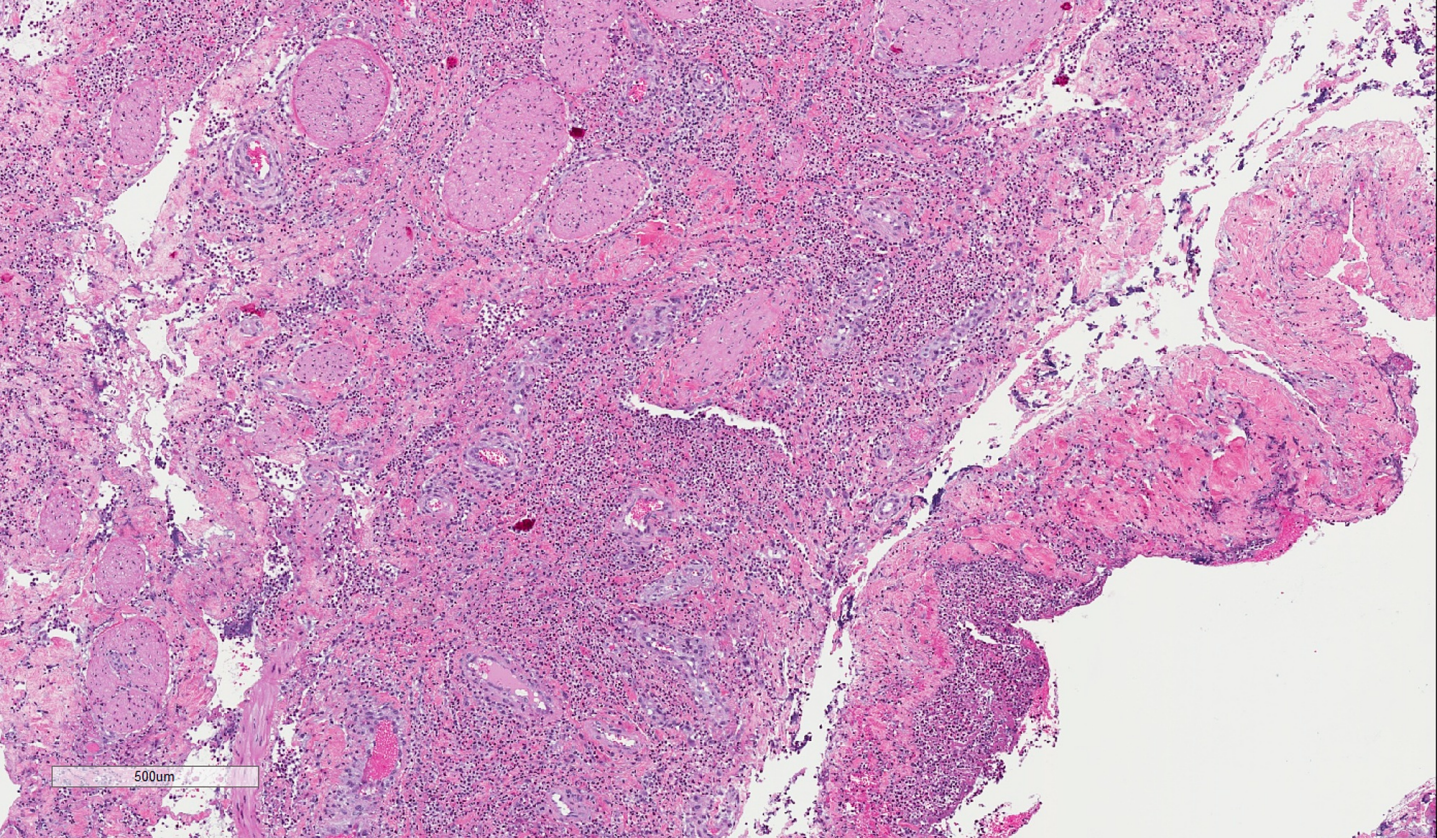
Scrotum- Diffuse dermal neutrophil rich inflammation with suppuration, extending into the underlying muscle and fascia

On day 142 the patient presented with an erythematous excoriated scrotum and tender, hard, right testis. No further spread of cellulitis was observed. The abdomen was soft, non-tender, with a clean wound. An ultrasound scan suggested epididymo-orchitis without evidence suggestive of abscess formation or necrotizing infection. Derangement of blood investigations (Table 1) and further systemic deterioration resulted in emergency debridement. Intraoperatively extensive infected tissue necrosis including subcutaneous tissue and dartos fascia was found and resected (Figure 2). A diagnosis of NTSI of the scrotum was made, also known as Fournier’s gangrene. During this 28-day admission microbial wound swabs, tissue, urine, blood, and faecal cultures showed no growth. This was likely due to sustained broad-spectrum antibiotic therapies during his disease course. Delayed reconstruction of the scrotum was performed using a split skin graft. The patient was discharged on day 169 with return to near baseline and/or accepted normal parameters for all bloods (Table 1).

Throughout his hospitalization the patient received care from a plethora of specialist teams including general surgery, urology and plastic surgery for debridement and reconstruction. Intensive care followed post operatively. Infectious disease input for ongoing appropriate antibiotic choice, endocrinology for glycemic control, acute pain service for pain management, and medical teams for the management and optimization of existing comorbidities were involved. Prior to discharge the patient was cleared and deemed safe by a geriatric rehabilitation physician.

**Figure 3:**
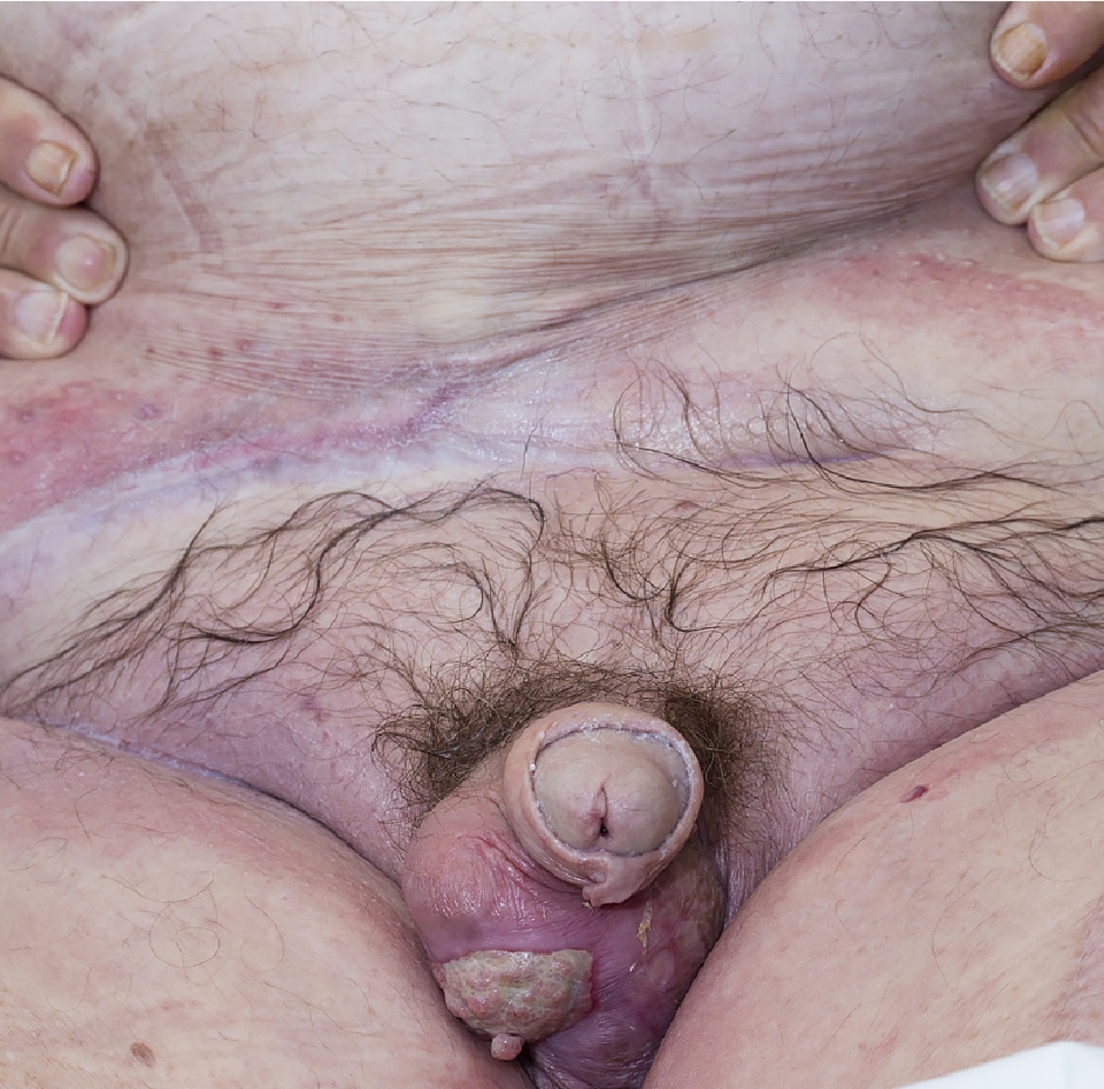
Scrotum, 90 days following split skin thickness graft. Abdominal wound 249 days after initial presentation.

Ongoing outpatient wound care and follow-up continues to the present day (Figure 3). At day 249 the patient is living independently and has a reasonable quality of life.

## Discussion

This case illustrates both an indolent course of what may be described as a form of sub-acute abdominal wall NSTI/necrotizing fasciitis as well as a hyperacute presentation of a fulminant NSTI in the form of a Fournier’s gangrene.

Necrotizing soft tissue infections are a very uncommon though deadly disease. For Fournier’s gangrene, a subtype affecting scrotum and the perineal region, frequently an incidence of 1.6/100,000 population wide is reported with peak incidence in the age group above 50 at 3.3/100,000 [8]. Mortality for NSTIs ranges from 20-40% in mostly small retrospective series [8-10]. An American nationwide retrospective epidemiological investigation of Fournier’s gangrene by Sorensen et al. of 1641 patients across two calendar years demonstrated a pooled mortality of 7.5% but in some individuals up to 88% [8].

Accurate diagnosis of NSTI is difficult due to the absence of easily distinguishable signs that separate it from other cutaneous infections [4-5,11-13]. An accurate diagnosis of NSTIs on admission has been reported at 15-30% [5]. Most cases are misdiagnosed as erysipelas, cellulitis, and abscess of the affected area. Clinically distinguishing cutaneous features include pain disproportionate to overt extent of disease, no clear demarcation of erythema, and absence of lymphangitis. These are signs associated with erysipelas and cellulitis respectively [4-5,11-13].

Given the paucity of systemic symptoms and inherent issues with accurate diagnosis, NSTIs may be sub-optimally managed for an extended time. This is apparent in the first presentation of this patient, who was initially managed as an abdominal pannus intertrigo, then cellulitis and then abscess. This disease picture is commensurate with the literature’s description of the sub-acute form of NSTI [4-7]. This form of the disease may progress over days to weeks without systemic compromise and may be indistinguishable from cellulitis [4,6]. Some authors have theorized that use of antibiotics on these sub-acute NSTIs may have minimal effect on the primary site of infection and could delay diagnosis as this impedes the development of systemic symptoms. Wong and Wang proposed diagnostic criteria to distinguish sub-acute NSTIs from the more acute variety: indolent and absence of systemic signs, gradual necrosis, progression despite antibiotics, sudden deterioration to overt NSTI with systemic features, and histology consistent with necrotizing infection [7]. Once systemic signs and organ compromise become evident, the disease course becomes indistinguishable from that of acute NSTI [5-7]. All these features were observed in the first presentation of this patient.

The pathophysiology of NSTI centers on the loss of skin integrity barrier mechanisms and conditions predisposing to impaired host immunity [12]. This allows entry of the offending organism(s). NSTI may be polymicrobial or monomicrobial in nature [1,2,14]. The infection moves through the deeper layers of the soft tissues, along the fascial planes, hence given the name of necrotizing fasciitis, a vigorous immune response subsequently occurs with extensive neutrophil rich inflammation [15]. An ensuing angio-thrombotic microbial invasion of fascia and obliterative endarteritis progresses to cause necrosis of the dermal and subcutaneous tissues [5,13,16-17]. This explains how signs may be minimal with disproportionate pain as the infection initially proliferates in the deeper layers of the soft tissue [2,5,11-12].

Diabetes mellitus is a risk factor in up to 70% of all NSTI patients, including the Fournier’s gangrene subtype [2,4,5,8,9,11-18]. Additional commonly described risk factors include increasing age, chronic liver disease, chronic kidney disease, obesity, immunosuppression, underlying malignancy, and poor hygiene [5,10,12,16]. This patient had many of the risk factors. Based on history alone, this warrants a higher clinical suspicion for NTSI.

In addition to these risk factors which may raise the index of suspicion for a diagnosis of NSTI, Wong et al. developed a Laboratory Risk Indicator for Necrotizing Fasciitis (LRINEC) Score [19]. This score may be used to risk-stratify patients with soft tissue infection as to whether NSTI is likely (Table 2). A score of higher than or equal to 6 indicates intermediate to high risk for NSTI. A score of 8 or above confers a greater than 75% risk of the diagnosis being NSTI [16-17]. This patient discussed had an LRINEC score of 10 on presentation with the anterior abdominal wall NSTI and a score of 12 on second presentation with Fournier’s gangrene (Table 1). This, in addition to his risk factors, made the diagnosis of NSTI highly likely and justified the emergent operating theatre access for this patient. It should be noted that the LRINEC score has not been validated for use in patients where NSTI is not apparent at initial investigation and a low-risk score should not be used for management decisions if a high clinical suspicion of NSTI still exists [5,11,19]. 

**Table 2:** Laboratory risk indicator for necrotizing soft tissue infections (LRINEC) scoring criteria [5,16,19]

Investigations:	Score
Serum C- Reactive Protein (mg/L)	
<150	0
≥150	4
Hemoglobin (g/L)	
>135	0
110-135	1
<110	2
White cell count (x10^9/L)	
<15	0
15-25	1
>25	2
Serum sodium (mmol/L	
≥135	0
<135	2
Serum creatinine (µmol/L)	
≤141	0
>141	2
Serum glucose (mmol/L)	
≤10	0
>10	1

Imaging may be a useful adjunct in the diagnosis of NSTIs but the rapid progression of the disease means that imaging should not delay definitive management [1,5,12,13,16]. Common findings in conjunction with clinical correlation that confer a high suspicion for NSTI occur in plain x-ray, ultrasound, CT, and MRI scans. X-ray may demonstrate soft tissue gas representing subcutaneous emphysema [1,5,12]. Ultrasound can identify soft tissue changes in NSTI [1]. “Dirty shadowing” may be observed due to soft tissue inflammation and subcutaneous gas as well as collections [12,15,16]. CT can identify gas and fluid tracking along fascial planes as well as fluid collections [5,12,13]. MRI may demonstrate deep fascial fluid collection with tissue thickening and enhancement after contrast administration [1,5,13].

An invasive diagnostic test that has been described is the “finger test”. This involves infiltrating the suspected area with local anaesthetic, making a finger width incision to the deep fascia. If the finger is inserted and can easily blunt dissect the subcutaneous tissue off the fascia, the test is said to be positive. Other macroscopic findings that may be observed include “dishwater fluid,” a watery, malodorous fluid collection among oedematous, necrosed tissues [11].

The literature consensus for management of all NSTI is centred on resuscitation of the patient to minimise end-organ damage, broad-spectrum antibiotics, and immediate, radical resection of affected tissue [1-5,8,9,11-18]. Time to surgery is often described as the most important determinant for reducing mortality and morbidity [5,12-15]. A delay in diagnosis and surgical debridement of both NSTIs generally as well as Fournier’s gangrene increases the area of tissue involvement and increases mortality [1,3,5,12,15]. An average of three to four debridements are required during the hospital stay for NTSIs [1]. This in conjunction with the length of hospital stay demonstrates the severity of the disease. Many authors describe the need for radical resection as being judicious, and attempting to conserve tissue may result in poorer outcomes [1-3,5,8-12,14-18].

Broad-spectrum antibiotics should cover *S. aureus* (including MRSA if locally indicated) and *S. pyogenes* as well as gram negative aerobes and anaerobes. NSTI are commonly polymicrobial [1,5,10,12,14]. Specific types of NSTI such as Fournier’s gangrene are likely to involve gram negative organisms, so the use of metronidazole has been suggested [1,10]. The principles described for antimicrobial management are commonly presented in the literature but no consensus for antimicrobial choice exists due to the rarity and severity of the disease. Local guidelines can differ [1,5,10,12,14].

**Figure 4:**
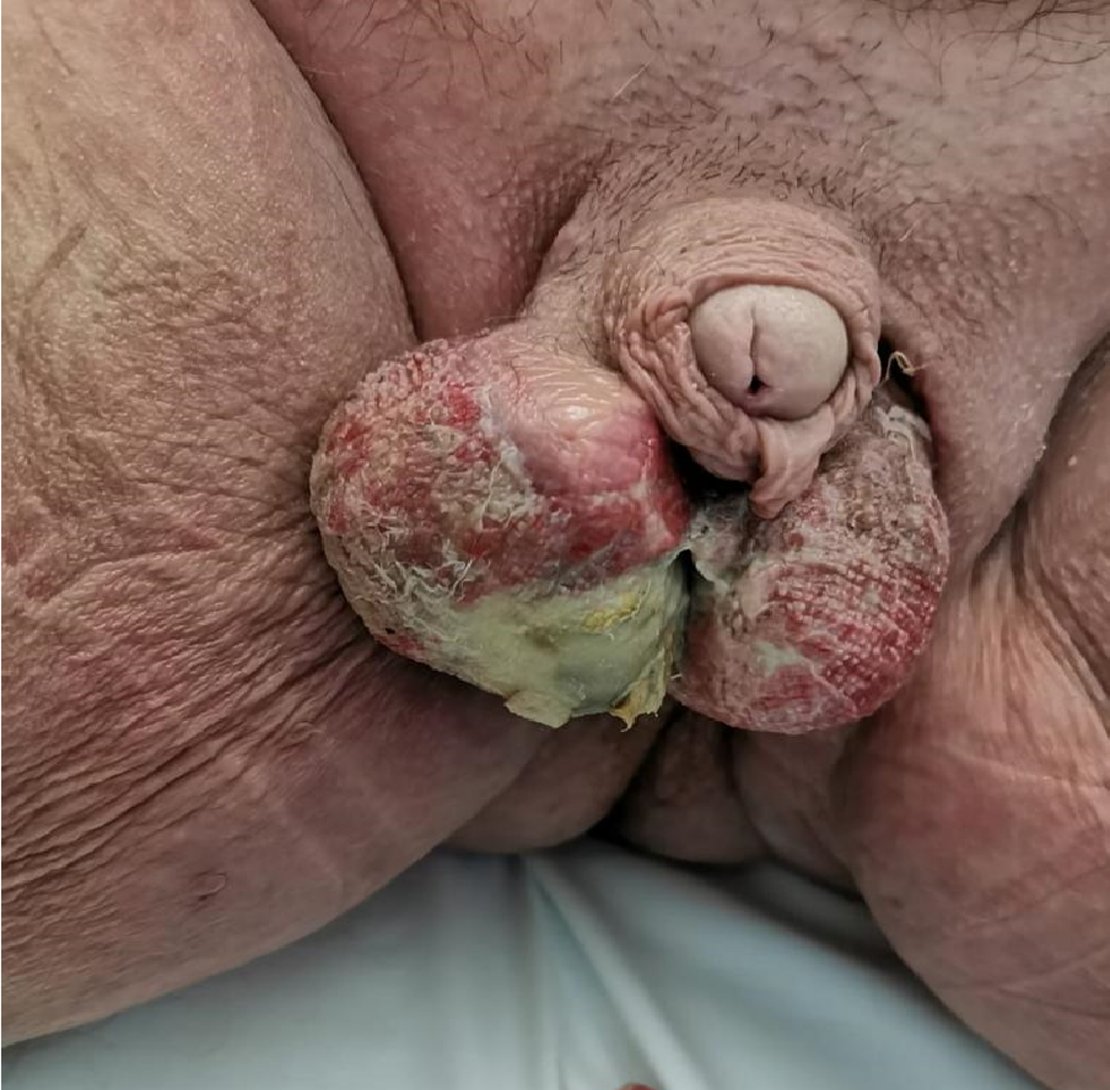
Scrotal defect after initial debridement and skin graft.

After initial debridement, negative pressure VAC dressing can increase granulation and reduce tissue edema preparing the wound bed for possible reconstruction [1,10,12,13,14,16]. The wounds of a debrided NSTI are not considered appropriate for a primary closure. In the second presentation of the patient, the Fournier’s gangrene, several reconstructive methods were considered: advancement flap, perforator, and muscle flaps [1,9,14,16-18]. These were all deemed inappropriate due to the poor health status of the patient. Evidence of good outcomes with split thickness skin graft led to this becoming the reconstruction method of choice for the scrotal skin deficit (Figure 4) [20].

Not only are there high mortality and morbidity for NSTIs, but also lengthy hospital stays and frequent requirement for multidisciplinary management results in high cost for care. In the large epidemiological study by Sorensen, on discharge 30% of patients required home health care or access to a skilled nursing facility [8]. Even now at day 249 (Figure 3) with his remarkable recovery and independence in daily activities, the patient still requires ongoing wound care.

## Conclusions

Necrotizing soft tissue infections present a diagnostic dilemma to family physicians and emergency departments alike. They have a very low incidence, high mortality, and potential for rapid deterioration. In particular, the sub-acute form of NSTI may be indistinguishable from cellulitis and antibiotic management could mask constitutional signs and symptoms. A high index of suspicion should exist in patients with comorbidities that reduce barrier mechanisms and host immunity. This in correlation with exam findings, blood investigations, and imaging can clinch the diagnosis.

On presentation patients need resuscitation for the minimization of end-organ damage and broad-spectrum antibiotics. The only definitive management step is immediate and radical resection of the infected tissue.

NSTIs usually result in large tissue defects and plastic surgery consultation may be required to determine appropriate reconstruction. Furthermore, multidisciplinary input in addition to plastic surgery is often a requisite to provide appropriate care for these very sick patients. On discharge many patients require prolonged rehabilitation and wound care.

Rapid recognition, immediate surgical intervention, and specialist care are required to reduce the mortality and morbidity associated with NSTIs.
